# microRNA-338-3p suppresses lipopolysaccharide-induced inflammatory response in HK-2 cells

**DOI:** 10.1186/s12860-022-00455-0

**Published:** 2022-12-23

**Authors:** Jing Wang, Guokai Li, Min Lin, Sheng Lin, Ling Wu

**Affiliations:** 1 Department of nosocomial infection management, Fujian Maternity and Child Health Hospital, Fujian Fuzhou, 350001 China; 2 Pediatric intensive care unit, Fujian Maternity and Child Health Hospital, Fujian Fuzhou, 350001 China; 3 Department of pediatrics, Fujian Maternity and Child Health Hospital, No. 18 Daoshan Road, Gulou District, Fujian Fuzhou, 350001 China

**Keywords:** MicroRNA, Inflammation, Kidney injury, Lipopolysaccharide, Mitochondrial membrane potential

## Abstract

**Background:**

Inflammation is the most common cause of kidney damage, and inflammatory responses in a number of diseases are mediated by microRNA-338-3p (miR-338-3p). However, there are only a few reports which described the regulation of miR-338-3p in human proximal tubular cells. The goal of this study was to see how miR-338-3p affected lipopolysaccharide (LPS)-caused inflammatory response in HK-2 cells.

**Methods:**

LPS was used to construct an inflammatory model in HK-2 cells. miR-338-3p mimic was used to increase the levels of miR-338-3p in HK-2 cells. MTT, JC-1 staining, and apoptosis assays were used to detect cell viability, mitochondrial membrane potential (MMP), and apoptosis, respectively. The production of inflammatory factors and the levels of p38, p65, phospho-p65, phospho-p38, Bax, Bcl-2, cleaved caspase-9, and cleaved caspase-3 were investigated using real-time polymerase chain reaction, western blotting, or enzyme-linked immunosorbent assay.

**Results:**

The levels of miR-338-3p were significantly lower in serum from patients with sepsis-induced kidney injury compared to the serum from healthy volunteers (*P* < 0.05). LPS reduced the level of miR-338-3p in HK-2 cells (*P* < 0.05). HK-2 cell viability, mitochondrial membrane potential, and Bcl-2 mRNA and protein levels were decreased by LPS (all *P* < 0.05). Apoptosis, the mRNA and protein levels of inflammatory cytokines (IL-1β, IL-6, IL-8, and TNF-α) and Bax, and the levels of cleaved caspase-9 and caspase-3 were increased by LPS (all *P* < 0.05). Raising the level of miR-338-3p mitigated these effects of LPS (all *P* < 0.05).

**Conclusion:**

LPS-induced inflammation in HK-2 cells is reduced by miR-338-3p.

**Supplementary Information:**

The online version contains supplementary material available at 10.1186/s12860-022-00455-0.

## Introduction

Sepsis is caused by the dysfunctional response of the host to infection, which produces a large number of inflammatory factors. Acute kidney injury (AKI) is a common complication among hospitalized patients with sepsis, is associated with increased mortality [1, 2], and is characterized by endothelial injury with hemodynamic dysfunction [3]. Inflammatory cytokines (IL-1β, IL-6, IL-8, TNF-α) produced by the proximal tubular epithelial cells of the damaged kidney, penetrate into the renal interstitium and further damage renal function; the resultant damage indicates that the pathogenesis of AKI is complicated by inflammation [4–6]. Oxidative stress, together with inflammation, can accelerate the decline of renal function [7, 8]; therefore, the exploration of inflammatory responses in renal injury will contribute to the treatment and improve the survival rate of patients with this condition. The NF-κB (p65) and MAPKs (p38) signaling pathways are the main pathways that regulate the production of inflammatory cytokines [9], so the activation of these pathways will improve the production of inflammatory cytokines. Furthermore, sepsis-induced AKI is considered to be a comprehensive response, including transcriptional events, mitochondrial activity, and apoptosis [3].

MicroRNAs (miRNAs) are non-coding RNAs containing approximately 20–24 nucleotides that target the 3’-untranslated region of messenger RNA (mRNA) to regulate its degradation or translation [10, 11]. miRNAs affect the development of inflammatory diseases, according to mounting evidence [6, 12–14]. For example, miRNA-221 and miRNA-222 inhibit endothelial cell proliferation and angiogenesis in chronic inflammation [8], whereas by targeting C-Myc in HK-2 cells, miRNA-103 aggravates the inflammatory damage caused by lipopolysaccharide (LPS). Additionally, miRNA-30b enhances LPS-induced inflammatory damage in HK-2 cells, and promotes the production of inflammatory cytokines, and these processes lead to cell dysfunction [6, 12, 15]. Another example is miRNA-500 A-3p, which has anti-inflammatory properties that can alleviate renal injury [16].

MiRNA-338-3p (miR-338-3p) is located in the seventh intron of the apoptosis-associated tyrosine kinase gene [17]. Apoptosis is the main mode of cell death, the decrease of mitochondrial membrane potential is the early activity of apoptosis, and the dysregulation of apoptosis-related proteins (Bcl-2, Bax, cleaved caspase-9, and cleaved caspase-3) plays an important role in the occurrence and development of apoptosis [18]. Therefore, the investigation of the relationship between miR-338-3p and apoptosis-related proteins (Bcl-2, Bax, cleaved caspase-9, and cleaved caspase-3) is helpful to reveal the molecular mechanism of miR-338-3p’s regulation of inflammatory damage in renal cells. The level of miR-338-3p is decreased in virus-induced neurodegenerative diseases [19]. miR-338-3p can alleviate inflammatory damage. For example, miR-338-3p inhibits inflammation in acute liver injury caused by N-acetyl-p-aminophenol, and relieves LPS-induced inflammatory damage in 16HBE cells (human bronchial epithelioid cells) [20, 21]. However, it is unknown what role miR-338-3p plays in renal injury.

In this study, HK-2 cells were stimulated by LPS to establish an in vitro model of inflammatory damage. HK-2 cells overexpressing miR-338-3p were treated with LPS, and then cell viability, the secretion of inflammatory cytokine, mitochondrial membrane potential (MMP) changes, and apoptosis were detected to study the role of miR-338-3p in LPS-induced inflammation.

## Materials and methods

### Cell culture

The HK-2 cell line, which is an epithelial cell line of the proximal convoluted tubule of the human renal cortex, was purchased from Xiamen Immocell Biotechnology Co., Ltd. (Catalog number: IM-H060). DMEM with 10% fetal bovine serum and 5ng/ml epidermal growth factor was used to culture the cells. The cells were incubated at 37 °C, with 5% carbon dioxide, and 70–80% humidity.

### Grouping of cells

Dimethyl sulfoxide (DMSO), LPS, LPS + mimic negative control (NC), and LPS + miR-338-3p mimic groups designed with HK-2 cells in 6-well plates. In the DMSO group, HK-2 cells were treated with complete medium supplemented with 0.1% DMSO (as a negative control) for 24 h. In the LPS group, HK-2 cells were treated with 5 µg/mL LPS for 24 h. LPS + mimic NC group and LPS + miR-338-3p mimic group were transfected with 200 pmol negative control of miR-338-3p mimic (mimic NC) and miR-338-3p mimic for 24 h, respectively, and then the cells were treated 5 µg/mL LPS. After 24 h, cells or cell supernatants were collected for subsequent experiments.

### Blood sample collection

The Fujian Maternity and Child Health Hospital Ethics Committee approved this study (approval number: 2022KD0133), which was carried out in compliance with the Helsinki Declaration. Informed consent papers were signed by all volunteers. Vacuum vascular collection was used to collect peripheral venous blood (5 mL) from all volunteers in the morning while they were fasting. The blood was centrifuged to isolate the serum which was stored at -80 °C for further analysis.

### Real-time polymerase chain reaction (RT-PCR)

To extract RNA, cells were treated with TRI reagent® (Sigma-Aldrich, Catalog number: T9424) and serum was treated with serum miRNA isolation kit (TIANGEN, Catalog number: DP503) according to the manufacturer’s instructions. The obtained RNA was reverse transcribed with the PrimeScript RT Reagent Kit (Takara, catalog number: RR047A). Reverse transcription of the miRNAs was completed using specific primers (Table 1). RT-PCR was performed using the reverse transcriptional RNA, Agilent-Strata gene MxReal-Time qPCR system, and SYBR Green Master Mix (VAZYME, catalog number: Q111-02). The thermocycling conditions of qPCR were 95 °C for 5 min, followed by 40 cycles at 95 °C for 10 s and 60 °C for 30 s. The relative expression levels of genes were normalized to the 18 S rRNA levels using the 2^−ΔΔCq^ method. The primers used for RT-PCR are shown in Table 1.

**Table 1:** Primers for RT-PCR

Name	Sequence (5′-3′)	miRbase/Gene ID
miR-326-RT	GTCGTATCCAGTGCAGGGTCCGAGGTATTCGCACTGGATACGACCTGGAG	MIMAT0000756
miR-326-F	CGCCTCTGGGCCCTTC	
miR-126-5p-RT	GTCGTATCCAGTGCAGGGTCCGAGGTATTCGCACTGGATACGACCGCGTA	MIMAT0000444
miR-126-5p-F	GCGCGCATTATTACTTTTGG	
miR-338-3p-RT	GTCGTATCCAGTGCAGGGTCCGAGGTATTCGCACTGGATACGACCAACAA	MIMAT0000763
miR-338-3p-F	CGCGTCCAGCATCAGTGATT	
miR-599-RT	GTCGTATCCAGTGCAGGGTCCGAGGTATTCGCACTGGATACGACGTTTGA	MIMAT0003267
miR-599-F	CGCGCGGTTGTGTCAGTTTA	
miR-548 m-RT	GTCGTATCCAGTGCAGGGTCCGAGGTATTCGCACTGGATACGACCAAAAA	MIMAT0005917
miR-548 m-F	CGCGCAAAGGTATTTGTGG	
miR-16-5p-RT	GTCGTATCCAGTGCAGGGTCCGAGGTATTCGCACTGGATACGACCGCCAA	MIMAT0000069
miR-16-5p-F	CGCGTAGCAGCACGTAAATA	
miR-214-5p-RT	GTCGTATCCAGTGCAGGGTCCGAGGTATTCGCACTGGATACGACGCACAG	MIMAT0004564
miR-214-5p-F	CGCGTGCCTGTCTACACTTG	
miR-30c-5p-RT	GTCGTATCCAGTGCAGGGTCCGAGGTATTCGCACTGGATACGACGCTGAG	MIMAT0000244
miR-30c-5p-F	GCGCGTGTAAACATCCTACACT	
miR-221-3p-RT	GTCGTATCCAGTGCAGGGTCCGAGGTATTCGCACTGGATACGACGAAACC	MIMAT0000278
miR-221-3p-F	CGCGAGCTACATTGTCTGCTG	
miR-4763-3p-RT	GTCGTATCCAGTGCAGGGTCCGAGGTATTCGCACTGGATACGACCCCGCC	MIMAT0019913
miR-4763-3p-F	GCAGGGGCTGGTGCTG	
Bcl-2-F	ATCGCCCTGTGGATGACTGAGT	596
Bcl-2-R	GCCAGGAGAAATCAAACAGAGGC	
Bax-F	TCAGGATGCGTCCACCAAGAAG	581
Bax-R	TGTGTCCACGGCGGCAATCATC	
U6-F	CTCGCTTCGGCAGCACA	26,827
U6-R	AACGCTTCACGAATTTGCGT	
public reverse primer for miRNA	AGTGCAGGGTCCGAGGTATT	
18 s-F	ACCCGTTGAACCCCATTCGTGA	100,008,588
18 s-R	GCCTCACTAAACCATCCAATCGG	
*F* Forward primer, *R* Reverse primer, *RT* Specific primer of reverse transcription		

### MTT assay

To explore the effects of LPS and miR-338-3p on cell viability, we used MTT assay to detect cell viability. The treated HK-2 cell was seeded in 96-well plate at 1 × 10^4^ per well. After 12 h, 5 mg/mL MTT (10 µL per well) was added and incubated at 37 °C for 4 h. Then, we discarded the culture medium and added 150 µL DMSO to each well. The SpectraMax Absorbance Reader (Molecular Devices, San Francisco, CA, USA) was used to measure the absorbance at 490 nm.

### Western blotting

Protein was extracted using ice-cold RIPA buffer (Beyotime, Catalog number: P0013C). After quantification using the BCA protein concentration determination kit (Beyotime, catalog number: P0012S), the protein was separated by gel electrophoresis. The protein was then transferred to a PVDF membrane (Millipore, catalog number: IPVH00010), and incubated with 5% skim milk at 25 °C for 1 h. The protein-loaded PVDF membrane was incubated with BCL-2 antibody (catalog number: 12789-1-AP, Proteintech), BAX antibody (catalog number: 50599-2-Ig, Proteintech), GAPDH antibody (catalog number: 10494-1-AP, Proteintech), Cleaved Caspase-3 antibody (catalog number: ab32042, abcam), Cleaved Caspase-9 antibody (catalog number: ab2324, abcam), p65 antibody (catalog number: 10745-1-AP, Proteintech), p38 antibody (catalog number: 14064-1-AP, Proteintech), phospho-p38 antibody (catalog number: 28796-1-AP, Proteintech), or phospho-p65 antibody (catalog number: ab76302, abcam) at 4 °C overnight, followed by incubation with HRP-conjugated Affinipure Goat Anti-Rabbit IgG (catalog number: SA00001-2, Proteintech) at 25 °C for 1 h. After washed, the membranes were visualized by ECL chemiluminescence (Thermo Fisher Scientific).

### Detection of mitochondrial membrane potential (MMP)

After the treated cells were obtained, MMP was detected using JC-1 staining assay kit (Beyotime, catalog number: C2006) as directed by the manufacturer. The cells were then examined by flow cytometry (ACEA Bioscience Inc.) at Ex/Em = 549/575 nm.

### Apoptosis assay

Subsequent to treatment with the indicated compounds, HK-2 cells were collected to analyze apoptosis using an apoptosis detection kit (Vazyme, catalog number: A211-01) as directed by the manufacturer. Flow cytometry (ACEA Bioscience Inc.) was used to detect and analyze cell apoptosis.

### Enzyme-linked immunosorbent assay (ELISA)

The supernatant was collected after the treatments, and the IL-1β, IL-6, IL-8, and TNF-α levels were analyzed using Human IL-1 beta/IL-1F2 DuoSet ELISA Kit (R&D systems, catalog number: DY201-05), Human IL-6 Quantikine ELISA Kit (R&D systems, catalog number: D6050), Human IL-8/CXCL8 Quantikine ELISA Kit (R&D systems, catalog number: D8000C), or Human TNF-alpha Quantikine ELISA Kit (R&D systems, catalog number: DTA00D), as directed by the manufacturer. Finally, the protein concentration was determined with a microplate reader (Thermo Fisher Scien- tific, UK).

### Statistical analysis

The difference between two groups or among multiple groups was assessed using Student’s t-test (unpaired) or analysis of variance (ANOVA) in SPSS software (version 22.0), respectively. A difference of *P* < 0.05 was considered significant. GraphPad Prism 8.2.1 was used to obtain the graphs.

## Results

### LPS negatively regulates miR-338-3p

**Fig. 1:**
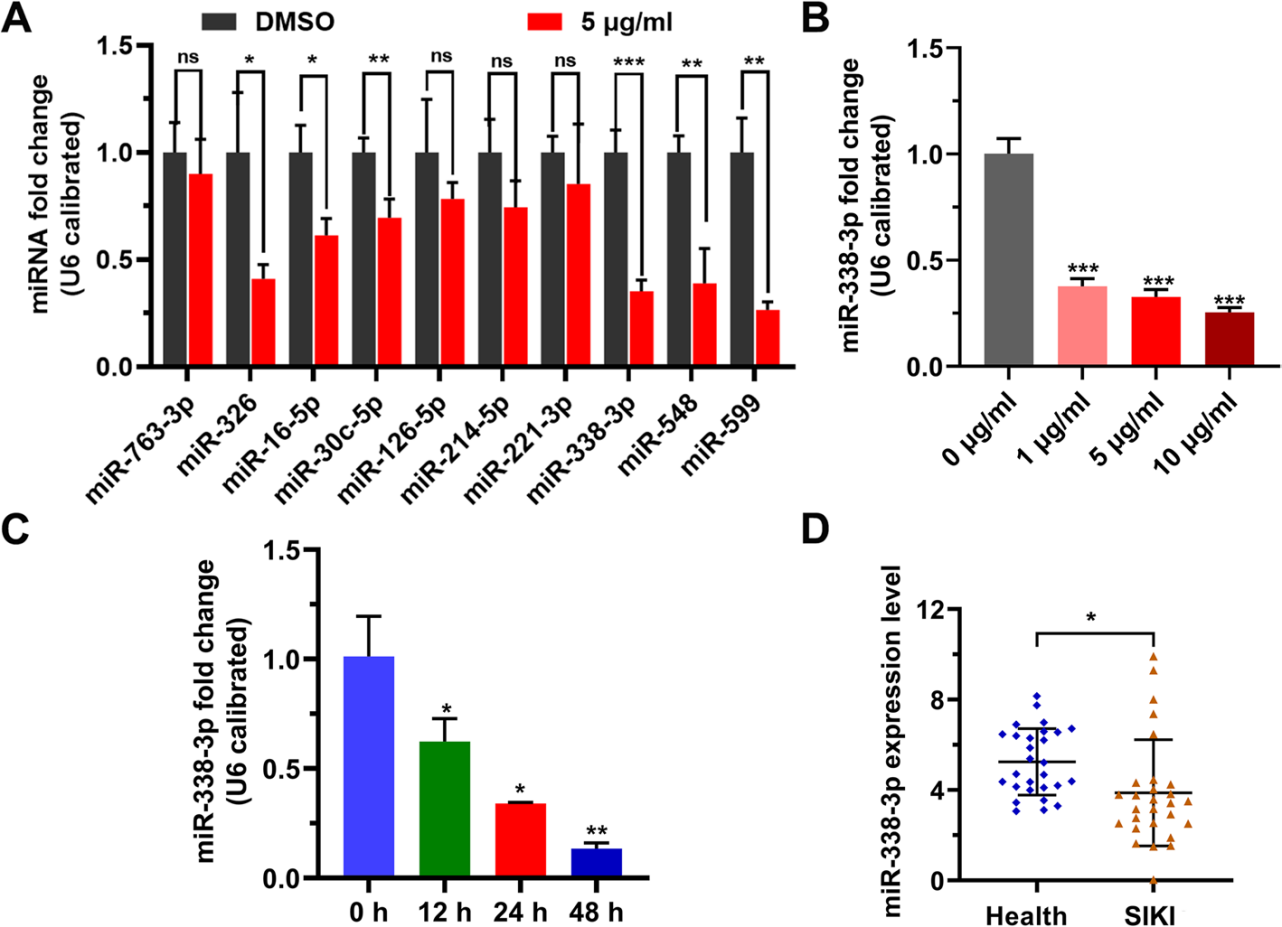
LPS decreases miR-338-3p level. **A**: The levels of 10 miRNAs in HK-2 cells stimulated by LPS for 24 h were tested by RT-PCR. **B**: miR-338-3p level in cells stimulated by LPS at different concentrations for 24 h was detected by RT-PCR. **C**: miR-338-3p levels in cells incubated by 5 µg/mL LPS for 12, 24, and 48 h were detected by RT-PCR. **D**: miR-338-3p level in the blood of patients with sepsis-induced acute kidney injury was determined by RT-PCR. SIKI, volunteers with sepsis-induced kidney injury; LPS, lipopolysaccharide. Ns: not significant, *: *p* < 0.05, **: *p* < 0.01, ***: *p* < 0.001

To investigate the effect of LPS on miRNA, we used 5 µg/mL LPS to stimulate HK-2 cells for 24 h, and RT-PCR was used to detect the levels of miRNAs in HK-2 cells. The results showed that in LPS-stimulated cells, the levels of miR-326, miR-16-5p, miR-30c-5p, miR-338-3p, miR-548, and miR-599 were significantly decreased (Fig. 1A). Subsequently, we discovered that the decrease of miR-338-3p in HK-2 cells occurred with the increase of LPS dose or the prolongation of stimulation time (Fig. 1B, C). Moreover, patients with sepsis-induced kidney injury had significantly higher levels of miR-338-3p in their blood than healthy volunteers (Fig. 1D). LPS inhibited miR-338-3p expression in HK-2 cells, according to these findings.

### LPS suppresses cell survival by targeting miR-338-3p

**Fig. 2:**
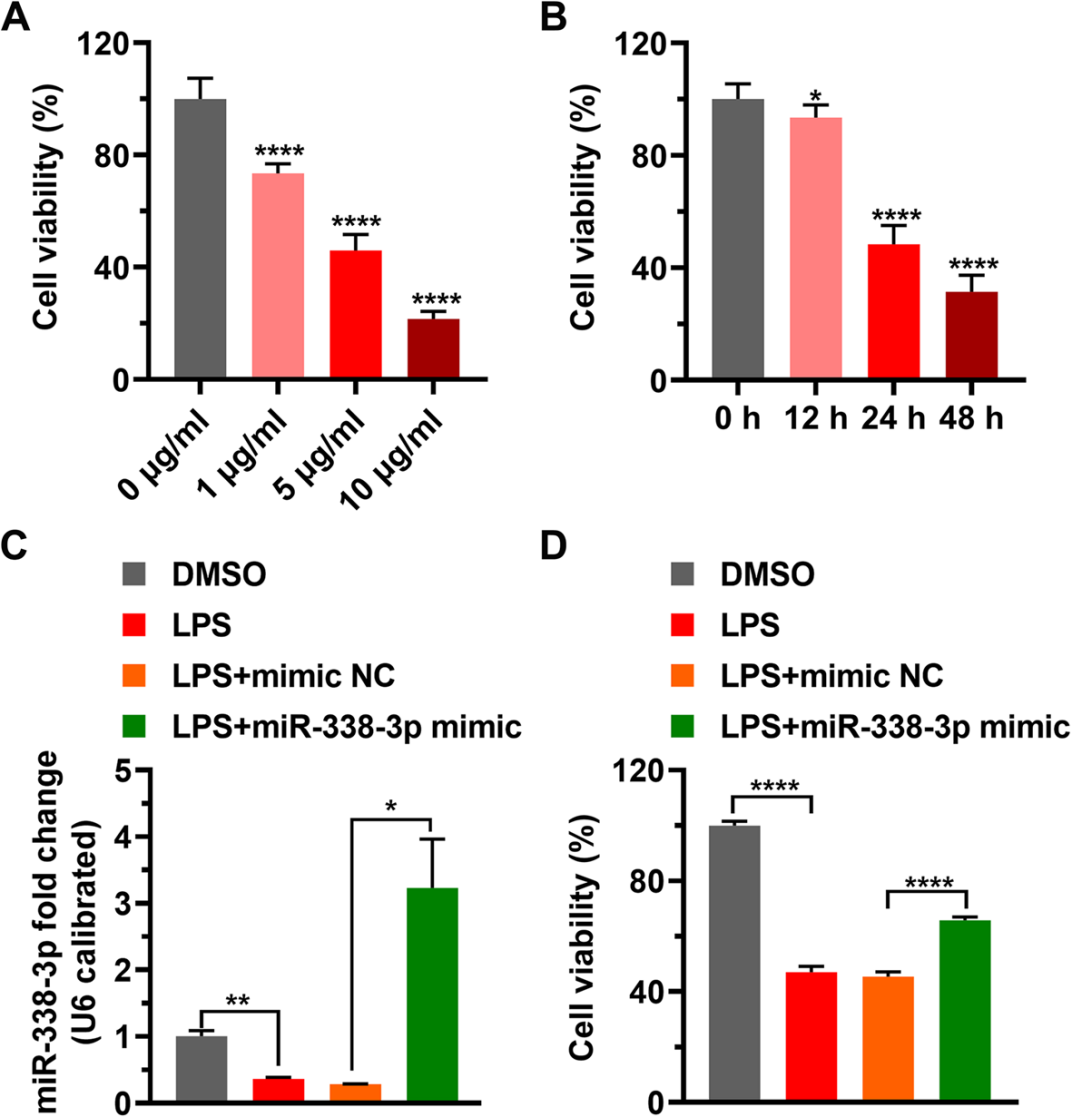
LPS inhibits HK-2 cell proliferation by targeting miR-338-3p. **A**–**B**: After incubation of HK-2 cells with various doses of LPS for 24 h, or with 5 µg/mL LPS for 12, 24, and 48 h, MTT assay was used to tested cell proliferation. **C**-**D**: After HK-2 cells were treated with DMSO, LPS, mimic NC, or miR-338-3p mimic, miR-338-3p level and cell proliferation were detected by RT-PCR and MTT assay, respectively. LPS, lipopolysaccharide; NC, negative control. *: *p* < 0.05, **: *p* < 0.01, ****: *p* < 0.0001

LPS affects the survival of cells by activating inflammatory response in the cells [6, 12]. The viability of HK-2 cells was determined using the MTT assay after they were stimulated with various concentrations of LPS or 5 g/mL LPS for various time periods. The results verified that the survival of cells was impaired by LPS, and the higher the amount of LPS, or the longer the LPS action time, the lower the survival rate of cells (Figs. 2A, B). The HK-2 cells were simultaneously treated with LPS and supplemented with miR-338-3p, and the levels of miR-338-3p and cell survival rate were detected. The results suggested that LPS reduced miR-338-3p level, but miR-338-3p level was increased when the HK-2 cells were treated with LPS and transfected with the miR-338-3p mimic (Fig. 2C). In addition, miR-338-3p diminished the LPS-induced decrease in cell survival (Fig. 2D). These data indicated that LPS impaired cell survival by inhibiting miR-338-3p expression.

### LPS induces inflammatory factor expression by reducing mir-338-3p level

**Fig. 3:**
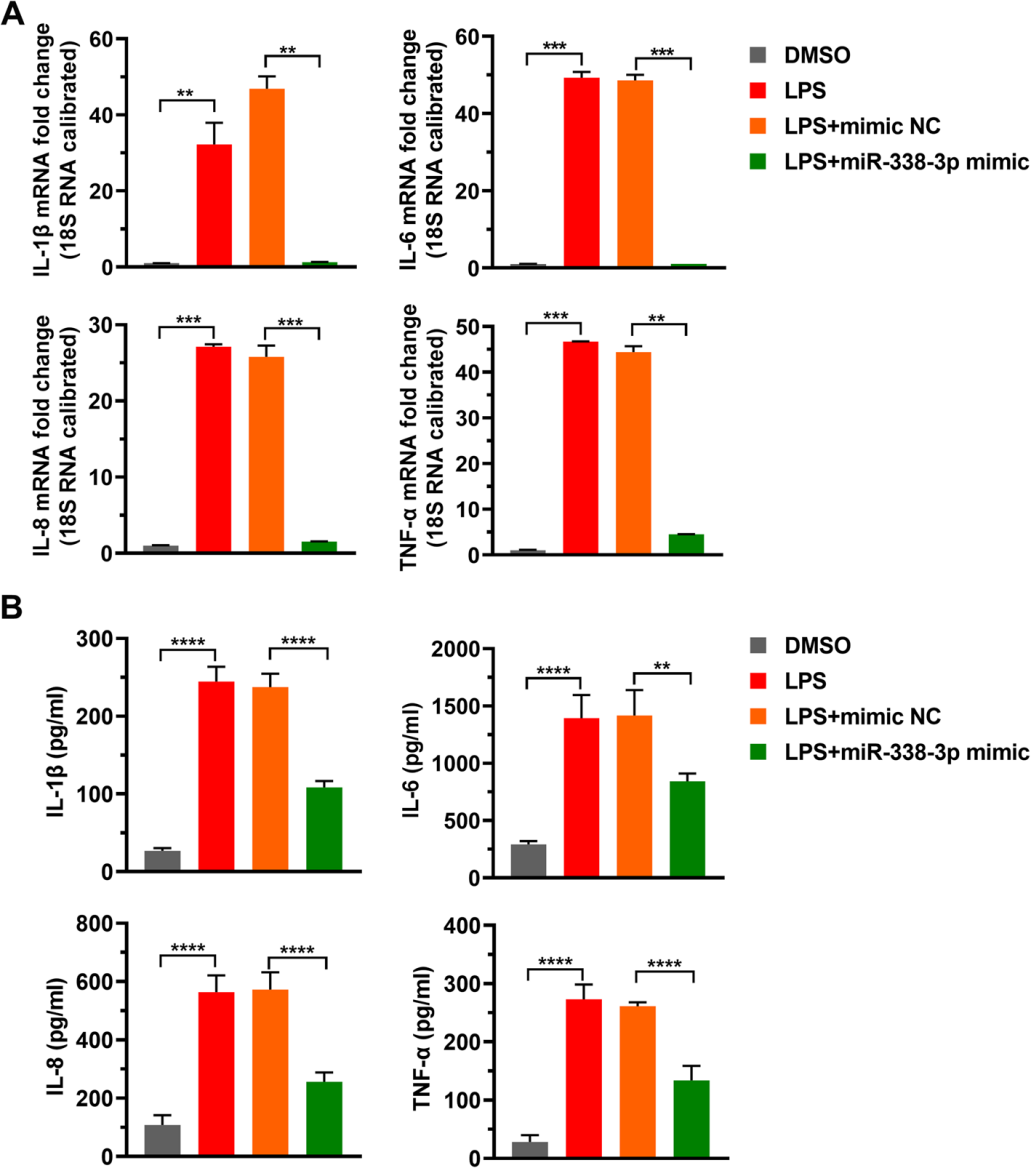
Increasing miR-338-3p level suppresses inflammatory factors’ expression induced by LPS. DMSO, LPS, LPS + mimic NC, or LPS + miR-338-3p mimic were applied to HK-2 cells. **A**-**B**: Inflammatory factors (L-1β, IL-6, IL-8, and TNF-α) mRNA and protein levels were tested by RT-PCR (**A**) and ELISA (**B**), respectively. LPS, lipopolysaccharide; NC, negative control. **: *p* < 0.01, ***: *p* < 0.001, ****: *p* < 0.0001

To explore whether LPS targeting miR-338-3p affects inflammatory factor expression, the LPS-treated cells were supplemented with miR-338-3p. We found that supplemented miR-338-3p alleviated LPS-induced expression of IL-1β, IL-6, IL-8, and TNF-α (Fig. 3). These findings implied that LPS induced the expression of inflammatory factors by targeting miR-338-3p.

### Overexpression of mir-338-3p relieves LPS-induced apoptosis

**Fig. 4:**
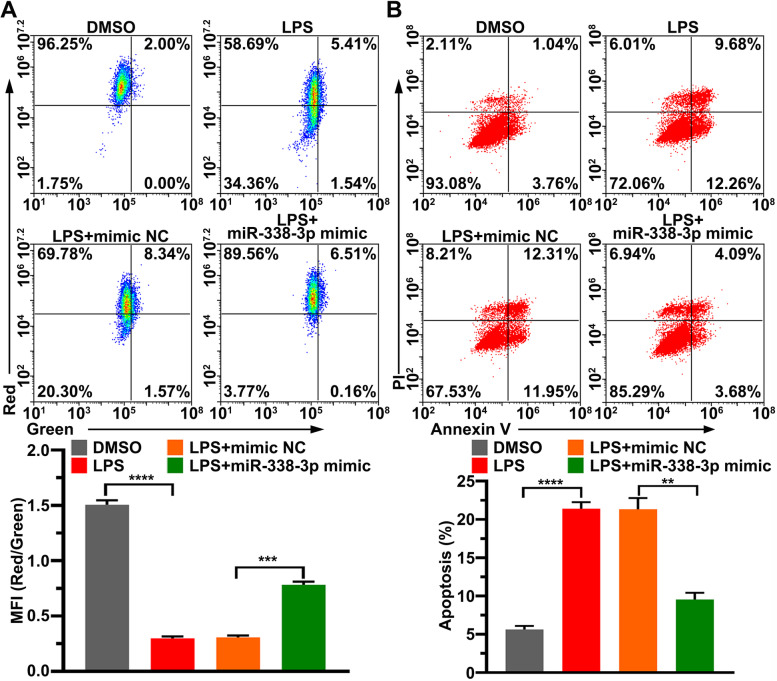
Elevated miR-338-3p alleviated LPS-induced apoptosis. **A**: MMP was detected by JC-1 staining. **B**: Annexin V-FITC/PI staining was used to gauge cell death. MMP, mitochondrial membrane potential; MFI, mean fluorescence intensity; PI, propidium iodide; LPS, lipopolysaccharide; **: *p* < 0.01, ***: *p* < 0.001, ****: *p* < 0.0001

**Fig. 5:**
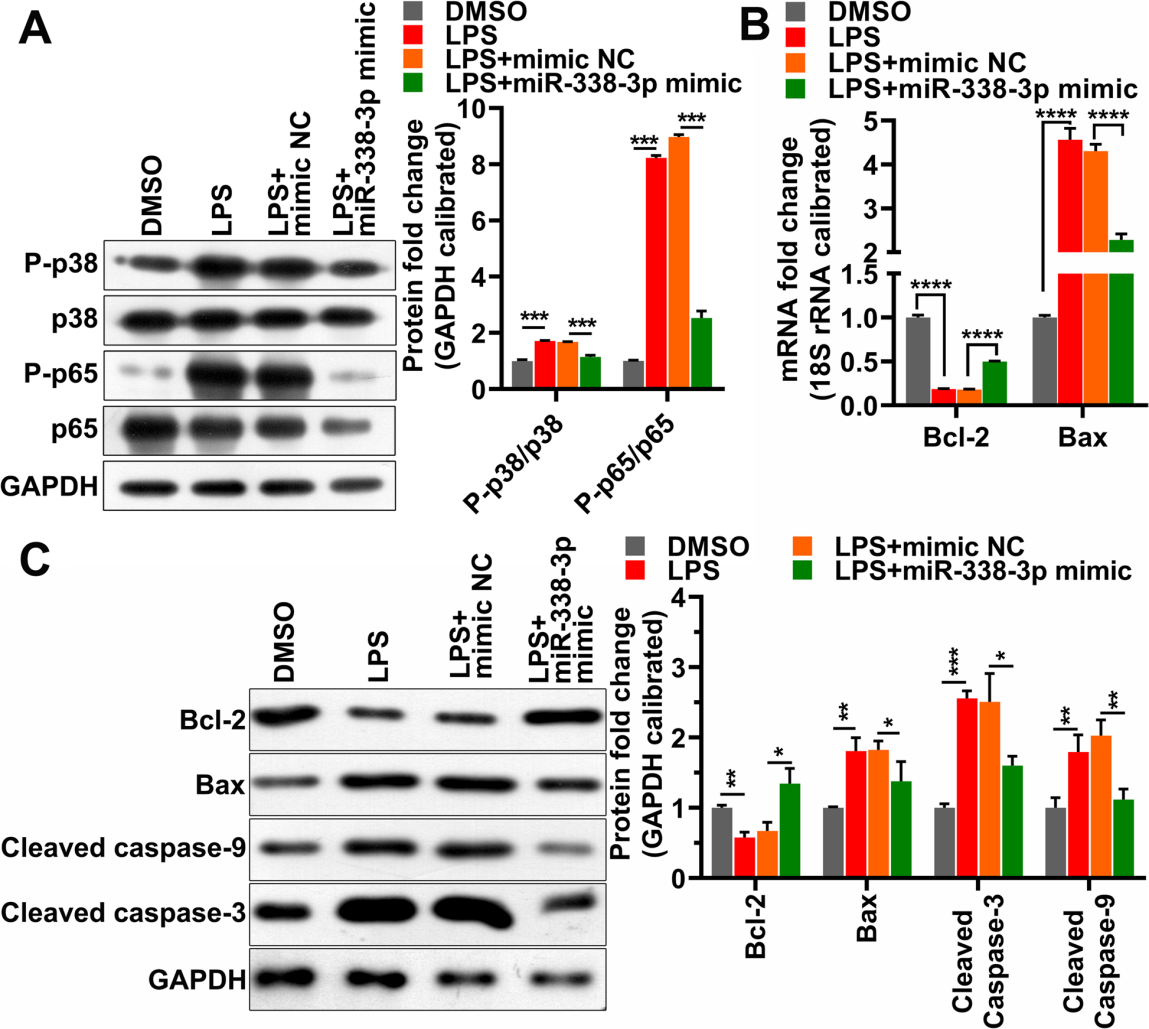
Overexpression of miR-338-3p inhibits LPS-induced phosphorylation of p65 and p38, as well as the regulation of apoptosis-related gene expression by LPS. **A**: Western blotting was used to test p65, p38, P-p65, and P-p38 levels. **B**: Bcl-2 and Bax mRNA levels were measured by RT-PCR. **C**: Western blotting was used to test Bcl-2, Bax, cleaved caspase-9 and cleaved caspase-3 protein levels. LPS, lipopolysaccharide; *: *p* < 0.05, **: *p* < 0.01, ***: *p* < 0.001, ****: *p* < 0.0001

According to previous report [22], LPS induces apoptosis, and the aforementioned results show that LPS targets miR-338-3p; therefore, it is possible that LPS promotes apoptosis by targeting miR-338-3p. We performed JC-1 staining to assess the changes in the MMP in HK-2 cells. The presence of green fluorescence indicates a decrease in MMP. As shown in Fig. 4A, LPS stimulation significantly reduced the MMP, while supplemented miR-338-3p alleviated the decrease in the MMP caused by LPS, indicating that LPS caused a decrease in the MMP by inhibiting miR-338-3p expression. Furthermore, an apoptosis assay showed that miR-338-3p’s overexpression reduced LPS-induced apoptosis (Fig. 4B). LPS promoted the phosphorylation of p65 and p38, while overexpression of miR-338-3p inhibited the LPS-promoted phosphorylation of p65 and p38 (Fig. 5A). LPS reduced Bcl-2’s mRNA and protein levels and increased Bax’s mRNA and protein levels, and enhanced cleaved caspase-9 and caspase-3 levels (Fig. 5B, C). In contrast to these results, the supplementary miR-338-3p eliminated these effects (Fig. 5B, C). These data suggest that LPS led to apoptosis by reducing miR-338-3p levels.

## Discussion

Sepsis is a systemic inflammatory response syndrome caused by infection that can lead to a variety of tissue and organ lesions, such as kidney damage and brain damage [23, 24]. Many miRNAs are associated with inflammation [14, 25]. In this study, to create an inflammatory injury model, we employed LPS to activate HK-2 cells. We observed that LPS caused HK-2 cells to become inflamed and die. We found that LPS damage in cell proliferation was reduced and the release of inflammatory cytokines was inhibited in HK-2 cells stimulated by LPS when miR-338-3p was overexpressed. Consistently, overexpression of MiR-338-3p has been shown to alleviate LPS-induced WI-38 cell damage [26]. In addition, LPS-induced apoptosis and reduced the MMP was alleviated when miR-338-3p level was increased. These data suggest that miR-338-3p has an anti-inflammatory effect in HK-2 cells.

Studies have shown that due to the role of miRNAs in regulating gene expression through targeted mRNA, miRNAs have regulatory effects on proliferation, apoptosis, autophagy, and inflammatory cytokines [12, 27]. Moreover, an increasing number of studies have shown that miRNAs can act as inflammatory response modulators [28, 29]. MiR-146a promotes the development of inflammation in CKD [30], and downregulation miR-223-3p and miR-93-5p in CKD led to stable increases in IL-6 and IL-8 [31]. Interestingly, miR-338-3p is implicated in the occurrence and progression of a variety of tumors, including breast, renal cell, cervical, colorectal, and lung cancers [32–40]. Some research has also linked miR-338-3p to inflammation in a variety of disorders. For example, miR-338-3p directly targets the *IKKβ* gene to regulate osteoclastogenesis, inhibits TNF-α-induced lipogenesis, and mitigates inflammatory damage induced by LPS in 16HBE cells [21, 41, 42]. Furthermore, miR-338-3p is associated with immune inflammatory responses in mice [43]. MiR-338-3p in the serum of patients with pancreatic cancer is correlated with the neutrophil count [44]. Here, we demonstrated that miR-338-3p has a role in the inflammatory response in HK-2 cells, and that LPS increases IL-1β, IL-6, IL-8, and TNF-α expression, and impairs cell proliferation by reducing miR-338-3p levels in cells.

The researchers looked at the impact of miR-338-3p on apoptosis and MMP changes in LPS-stimulated HK-2 cells. miR-338-3p promotes apoptosis by downregulating WNT2B expression in ovarian cancer cells [40]. However, we discovered that the LPS-induced apoptosis of HK-2 cells was assuaged when miR-338-3p was enhanced. These data imply that miR-338-3p has a dual function in apoptosis. Moreover, SW480 cell apoptosis is regulated by miR-338-3p, which targets MACC1 [45]. Downregulated miR-338-3p inhibits morphine-induced apoptosis by upregulating SOX4 expression and caspase-3-dependent apoptotic signaling pathways [46]. Nevertheless, our study shows that miR-338-3p mitigates apoptosis by reducing LPS-induced MMP reduction, Bax expression, caspase-9 and caspase-3 cleavage, and increasing Bcl-2 expression. Therefore, we conclude that miR-338-3p relieves LPS-induced cell proliferation damage by decreasing LPS-induced mitochondrial apoptosis.

Biological targets of miR-338-3p, such as RAB14, HIF-1, cyclinD1, ZEB2, PREX2a, and FOXP4, have been partially identified [32, 41, 47–49]. However, the precise molecular mechanism by which LPS targets miR-338-3p to induce apoptosis of HK-2 cells remains to be further studied. In addition, due to the lack of animal models of inflammatory kidney damage, the present study could not directly prove the role of miR-338-3p in inflammatory kidney injury.

## Conclusion

In summary, miR-338-3p alleviates inflammatory damage caused by LPS by regulating MMP, Bcl-2, Bax, P-p65, P-p38, and cleaved caspase-9 and caspase-3 levels. Thus, these results provide a new understanding of the pathological mechanism of inflammatory kidney injury and a theoretical basis for the treatment of this disease.

## Supplementary Information

**Additional file 1. **

## Data Availability

The datasets used and/or analyzed during the current study are available from the corresponding author on reasonable request.
