# Supplementary material for: microRNA-338-3p suppresses lipopolysaccharide-induced inflammatory response in HK-2 cells
Source: BMC Mol Cell Biol. 2022 Dec 23;23:60. doi: 10.1186/s12860-022-00455-0 (PMC9789656; doi:10.1186/s12860-022-00455-0)

Figure 4C: miR-338-3p decreased LPS-induced phosphorylation levels of p65 and p38.

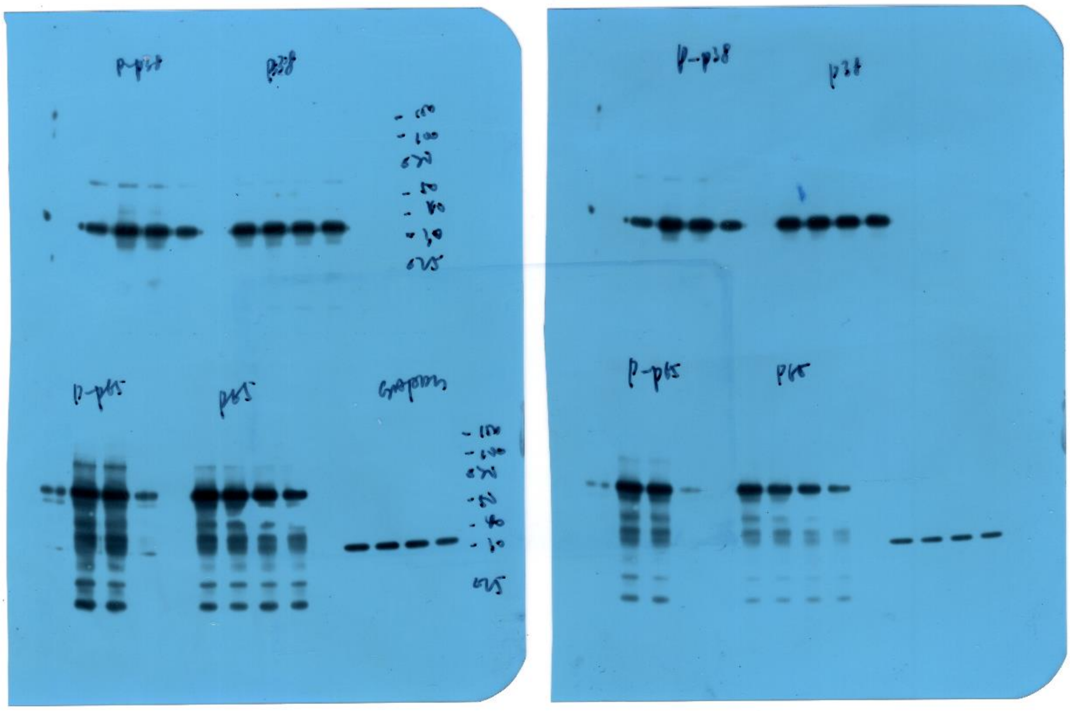

Figure 4E: miR-338-3p mitigated LPS-induced changes in Bcl-2 and Bax expression, as well as caspase-9 and caspase-3 cleavage levels.

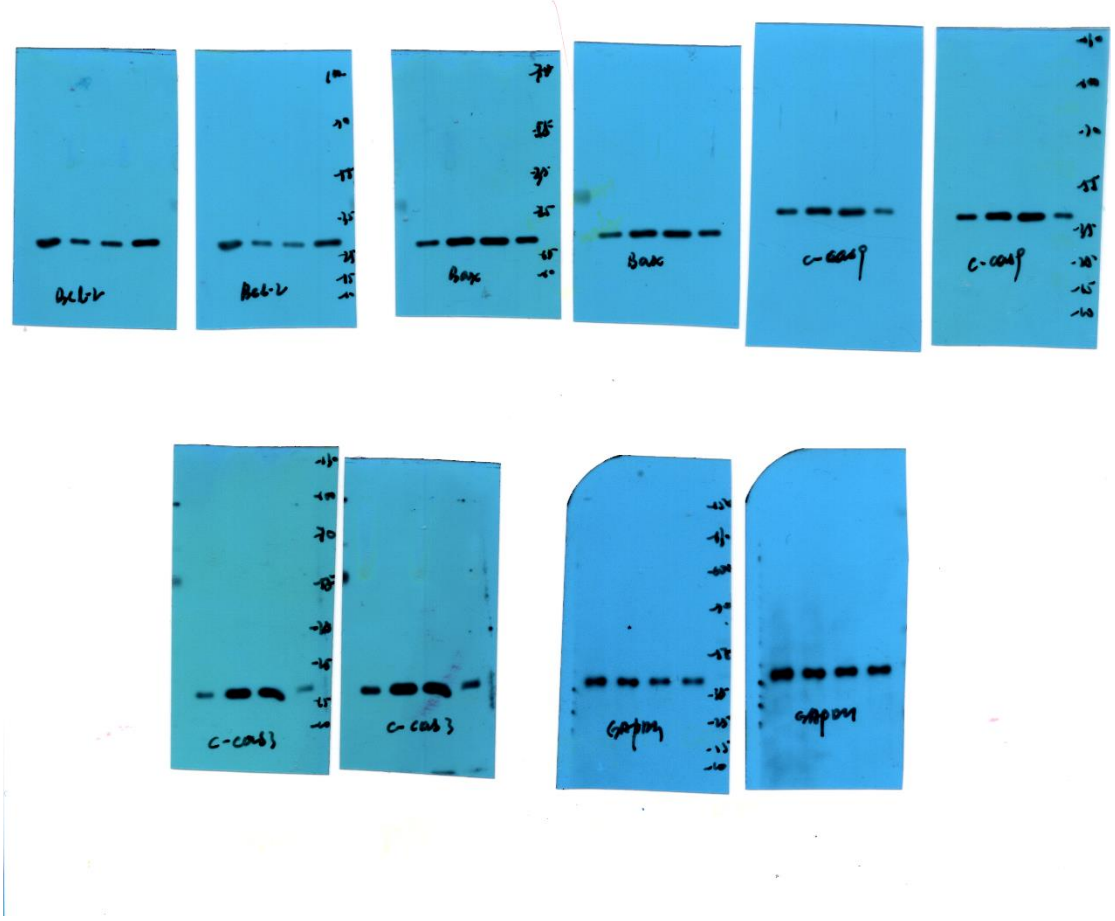

Supplement: Supplementary file 1 — Additional file 1. [file 12860_2022_455_MOESM1_ESM.pdf]
